# Effects of Antibiotic and Temperature Treatments on Phycosphere Bacterial Communities of Kelp Gametophytes

**DOI:** 10.1111/1758-2229.70425

**Published:** 2026-09-30

**Authors:** Yuewen Zhang, Shanshan Liang, Huiling Xia, Xiaoyu Zhong, Yi Zheng

**Affiliations:** ^1^ College of Life Science Fujian Normal University Fuzhou China; ^2^ Key Laboratory of Special Marine Bio‐Resources Sustainable Utilization of Fujian Province Fuzhou China

**Keywords:** gametophytes, phycosphere bacterial communities, *Saccharina japonica*

## Abstract

Microbiome dysbiosis represents a critical bottleneck in sustainable kelp aquaculture, yet the distinct regulatory mechanisms of antibiotics and temperature remain poorly characterized. This study systematically elucidates the divergent effects of penicillin–streptomycin exposure and temperature gradients on *Saccharina japonica* gametophytes and their epiphytic microbiota. Antibiotic treatment significantly reduced Proteobacteria from 93.02% to 49.70% and increased Bacteroidetes from 6.98% to 49.20%, replacing beneficial *Sulfitobacter* and *Colwellia* with opportunistic *Kordia* and *Klebsiella*, indicating severe dysbiosis driven by the creation of unoccupied ecological niches. In contrast, temperature variation did not alter dominant phyla: Proteobacteria remained predominant, but *Vibrio* peaked at 15°C (44.11%) alongside the maximal gametophyte specific growth rate of 5.21% per day, concurrently elevating pathogenic loads of *Vibrio* and *Pseudomonas*. These findings reveal that antibiotics disrupt core community architecture, whereas temperature modulates metabolic rates within stable taxonomic frameworks. Consequently, we propose a stratified preservation strategy: short‐term seedling cultivation at 15°C requires stringent antimicrobial measures to mitigate disease risk, while lower temperatures are preferable for long‐term germplasm storage to minimize contamination potential. This framework provides essential insights for precision microbial management, balancing growth promotion with disease control in kelp aquaculture systems.

## Graphical abstract

We reveal antibiotics drive qualitative turnover, while temperature induces quantitative fluctuations in kelp gametophyte microbiomes. This 15°C growth‐pathogen trade‐off guides precision seedling management.

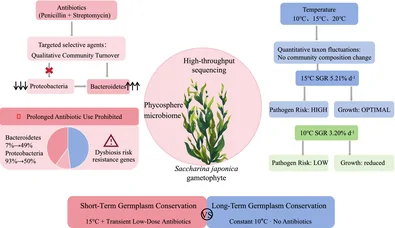

## 1 Introduction

*Saccharina japonica* represents an economically significant macroalgal species (Lee et al. 2005). This kelp undergoes a heteromorphic diplohaplontic life cycle characterized by alternating microscopic haploid gametophytic and macroscopic diploid sporophytic generations (Monteiro et al. 2019). The gametophytes, originating from germinated meiospores, lack tissue specialization and possess a simple body plan devoid of advanced defence or regulatory systems found in mature sporophytes (Wu et al. 2023; Zhou et al. 2022). This structural simplicity renders them highly susceptible to environmental fluctuations (Veenhof et al. 2023; Yu et al. 2023).

Successful germplasm preservation depends on maintaining a stable equilibrium between the host and its associated microbiota. However, bacterial contamination remains a persistent threat, with field‐derived materials often introducing opportunistic pathogens that compromise culture viability and germplasm integrity. Pathogenic infection interferes with the photosynthetic system of kelp, thereby indirectly leading to a reduction in the synthesis of carotenoids, such as fucoxanthin (Citti and Blanchard 2013), *Vibrio* infection may reduce fucoxanthin content by impairing photosynthetic metabolism. Meanwhile, *Pseudomonas* produce volatile sulphur compounds including dimethyl disulphide and dimethyl trisulfide at low temperatures, contributing to spoilage off‐odours in preserved samples (Parlapani et al. 2023). Antibiotic treatments are routinely employed to suppress pathogenic proliferation and stabilize gametophyte cultures (Sun et al. 2020). While effective in the short term, these interventions exert collateral effects by selectively eliminating susceptible bacterial taxa while permitting proliferation of resistant lineages, thereby restructuring the phycosphere community in ways that may undermine long‐term preservation stability.

The phycosphere microenvironment surrounding kelp gametophytes functions as a chemically defined niche shaped by algal exudation of dissolved organic matter and signalling metabolites (Bell and Mitchell 1972). The phycosphere microbiome associated with gametophytes exerts bidirectional regulatory effects on the host via metabolic exchange (Lian et al. 2018).

Temperature critically governs kelp gametophyte development by inducing sex‐biased transcriptional regulation (Monteiro et al. 2019), subsequently modulating phycosphere microbiota through thermal restructuring. Thermal shifts alter the competitive landscape within the phycosphere, favouring temperature‐adapted taxa while disrupting stable host‐microbe associations. Empirical observations indicate that gametophytes maintained at 15°C exhibit greater susceptibility to microbial contamination than those cultured at 10°C, suggesting that temperature regimes critically determine the stability of gametophyte‐microbiome interactions during preservation (Mars Brisbin et al. 2023).

Despite accumulating evidence that macroalgal microbiomes modulate host stress responses, bacterial dynamics in kelp gametophytes under preservation stress remain poorly resolved. Most studies focus on sporophytes, though Veenhof et al. (2025) recently showed that gametophyte and sporophyte microbiomes differ qualitatively and that gametophyte‐associated bacteria are essential for survival and thermal tolerance. Ocean warming can restructure kelp microbiota and enrich putative pathogens (Castro et al. 2024), and Molnar et al. (2025) demonstrated that temperature is a dominant driver of kelp microbiome composition. Yet the comparative effects of temperature fluctuations and antibiotic interventions—two routinely manipulated preservation variables—remain untested. High‐throughput sequencing has not been applied to resolve bacterial succession across preservation conditions in *Saccharina japonica* gametophyte systems (Vadillo Gonzalez et al. 2024). A central unresolved question is whether preservation stressors drive wholesale taxonomic turnover or merely adjust taxon abundances, a distinction critical for optimizing microbiome management. Clarifying these dynamics will advance both our understanding of early‐life‐stage holobiont interactions and evidence‐based germplasm preservation strategies.

Here, we applied this approach to track bacterial dynamics in *Saccharina japonica* gametophyte systems under paired temperature and antibiotic treatments during early gametophyte development. While prior work has separately documented each stressor's effects, no study has directly compared their regulatory outcomes or identified specific taxa linked to preservation risks. At 15°C, the gametophyte growth rate reached the maximum of 5.21% d^−1^, while the relative abundance of *Vibrio* also peaked at 44.11%, increasing the risk of pathogenic contamination. Concurrently, the relative abundance of *Pseudomonas* reached its maximum at this temperature. *Pseudomonas* can produce dimethyl disulphide and dimethyl trisulfide at low temperatures, leading to spoilage off‐odours. Antibiotic treatment reduced the proportion of Proteobacteria from 93.02% to 49.70%, while Bacteroidetes increased from 6.98% to 49.20%. This shift replaced beneficial genera such as *Sulfitobacter* and *Colwellia* with opportunistic pathogens such as *Kordia* and *Klebsiella*, thereby increasing pathogenicity. These findings provide taxon‐level evidence for microbial management in kelp germplasm preservation.

## 2 Materials and Methods

### 2.1 Experimental Materials

*S. japonica* reproductive stock was harvested from Lianjiang Guanwu Marine Science and Technology Development Co. Ltd., Fujian Province, China. Specimens were selected based on robust thallus architecture, uniform pigmentation, and absence of pathological signs. Microscopic inspection verified clean surfaces free of epibiotic colonization.

### 2.2 Experimental Procedures

#### 2.2.1 Zoospore Collection From Laminaria Breeding Stock

Mature *S. japonica* sporophytes meeting established morphological criteria were triple‐rinsed with 0.22 μm‐filtered sterile seawater. 5 × 5 cm explants were aseptically prepared using flame‐sterilized scalpels and surface‐cleaned with autoclaved gauze. Explants were immersed in 7‰ potassium iodide solution at 10°C for 20 min, then incubated at 12°C and 85% relative humidity with gentle aeration for 4 h. Treated tissues were transferred to 8°C sterile seawater to induce synchronous zoospore release.

#### 2.2.2 Establishment of Axenic Clonal Lines

Zoospore suspensions were diluted with seawater filtered through 0.22 μm polycarbonate membranes to 1–2 spores per microscopic field at 100× magnification using a hemacytometer. One millilitre aliquots were aseptically dispensed into 90 mm Petri dishes pre‐filled with 10 mL PES medium. Primary cultures were maintained at 10°C under continuous photosynthetically active radiation at 17–25 μmol photons·m^−2^ s^−1^ for 12 days in climate‐controlled growth chambers. Cultures were subsequently transferred to 1.7–2.5 μmol photons·m^−2^ s^−1^ and maintained for 15–20 days to induce gametogenesis.

Mature haploid gametophytes were sexually differentiated and individually isolated via micromanipulation at ×10 magnification using flame‐sterilized glass micropipettes. Isolated filaments were transferred to 50 mL autoclaved Erlenmeyer flasks containing enriched PES medium. Gametophytic aggregates were mechanically dissociated using a sterile tissue homogenizer at 2000 rpm with 30 s pulses to generate short filamentous segments. Resulting suspensions were washed three times with 0.22 μm filtered sterile seawater and passed through 200 μm nylon meshes to retain 2–5 celled fragments. Filtered fragments were re‐inoculated into fresh PES medium. Repeated subculture cycles produced axenic clonal lineages suitable for subsequent experimental scale‐up.

#### 2.2.3 Impact of Antibiotics on Phycosphere Communities in Kelp Gametophytes

Zoospore suspensions were diluted with seawater filtered through 0.22 μm polycarbonate membranes to 1–2 spores per microscopic field at ×100 magnification. One millilitre aliquots were aseptically dispensed into 90 mm Petri dishes pre‐filled with 10 mL Provasoli's Enriched Seawater medium. Experimental cultures received both penicillin (0.2 g/L) and streptomycin (0.1 g/L) simultaneously. Unsupplemented controls were maintained in parallel. All treatments were established in triplicate.

Cultures were incubated at 10°C under continuous photosynthetically active radiation at 17–25 μmol photons·m^−2^ s^−1^ for 12 d, after which irradiance was reduced to 1.7–2.5 μmol photons·m^−2^ s^−1^ for the remainder of the experimental period. Final sampling occurred on day 20. Planktonic bacterial cells were recovered from culture supernatants by vacuum filtration through 0.22 μm polycarbonate membranes and stored at −80°C pending metagenomic processing.

#### 2.2.4 Effects of Temperature on Phycosphere Communities in Kelp Gametophytes

Gametophytic clonal aggregates were mechanically dissociated for 90 s at 2000 rpm using a sterile tissue homogenizer. Homogenates were passed through 75 μm nylon mesh until 0.20 g of retained biomass was obtained. Exactly 0.20 g of this biomass was aseptically transferred to 250 mL Erlenmeyer flasks containing 150 mL sterile PES medium. All treatments were established in triplicate. Flasks were incubated in climate‐controlled growth chambers at 10°C ± 0.5°C, 15°C ± 0.5°C, and 20°C ± 0.5°C. Cultures were agitated orbitally at 180 rpm three times daily. Continuous photosynthetically active radiation was supplied at 17–25 μmol photons·m^−2^ s^−1^ for the first 12 d, after which irradiance was adjusted to 1.7–2.5 μmol photons·m^−2^ s^−1^ for the remainder of the 21 d experimental period.

Culture media underwent weekly replacement; depleted culture supernatants were vacuum‐filtered through 0.22 μm polycarbonate membranes for planktonic bacterial collection, with membranes cryopreserved at −80°C for subsequent metagenomic analysis. Epiphytic bacterial collection was conducted at the experimental endpoint (Day 21). Gametophyte biomass was aseptically harvested, rinsed briefly in sterile filtered seawater to remove loosely attached planktonic cells, and stored at −80°C pending DNA extraction. Bacterial cells were dislodged from algal surfaces via mechanical homogenization immediately prior to DNA extraction.

At terminal sampling (Day 21), 0.5 g fresh weight of female gametophytes from 10°C treatments was transferred to pre‐weighed centrifuge tubes and flash‐frozen in liquid N_2_ prior to −80°C storage. Post‐cultivation, all female gametophytes were harvested via 300‐mesh (48 μm) bolting silk filtration to gravimetric endpoint for biomass quantification. Specific growth rate (SGR; % day^−1^) was calculated as:

$$\mathrm{sK}=\frac{\ln W_{2}-\ln W_{1}}{t_{2}-t_{1}}\times 100\%$$

Variable definitions: sK, specific growth rate; *W*_1_, initial weight; *W*_2_, final weight; *t*_1_, start time; *t*_2_, end time.

#### 2.2.5 DNA Extraction and PCR Amplification

Genomic DNA was extracted from samples using the DNA Kit (Omega Bio‐tek, Norcross, GA, USA) following the manufacturer's instructions. The quality and quantity of extracted DNA were assessed by 1% agarose gel electrophoresis and spectrophotometric analysis. The V3‐V4 hypervariable regions of the bacterial 16S rDNA gene were amplified for microbial diversity analysis using the Illumina MiSeq PE300 platform. The primer sequences were as follows:

338F(5′‐ACTCCTACGGGAGGCAGCAG‐3′).

806R(5′‐GGACTACNNGGGTATCTAAT‐3′).

PCR amplification was performed in a total volume of 25 μL, comprising 12.5 μL KAPA 2G Robust Hot Start Ready Mix, 1 μL of each primer, 5 μL template DNA, and 5.5 μL nuclease‐free water. The thermal cycling protocol consisted of an initial denaturation at 95°C for 5 min; followed by 28 cycles of 95°C for 45 s, 55°C for 50 s, and 72°C for 45 s; and a final extension at 72°C for 10 min. Amplified products were purified using the GeneJET Gel Extraction Kit (Thermo Scientific, Waltham, MA, USA) according to the manufacturer's protocol.

#### 2.2.6 Data Quality Control and Biological Analysis

High‐throughput sequencing was conducted utilizing the Illumina MiSeq PE300 platform. Raw sequencing reads underwent initial quality control via adapter and primer excision employing Cutadapt. Chimeric sequences were subsequently subjected to bioinformatic filtration to generate refined effective datasets.

Bioinformatic analysis followed a standardized workflow. Raw sequencing reads were demultiplexed based on sample‐specific barcodes using Cutadapt. Sequences were clustered into operational taxonomic units at a 97% pairwise nucleotide identity threshold. Representative sequences for each operational taxonomic unit were taxonomically classified against a curated reference database, and abundance matrices were generated for all identified taxa. Community structure characterization included α‐diversity assessments covering species richness and Shannon evenness, as well as OTU abundance distribution analyses.

Data are expressed as mean ± standard error (*n* = 3). Raw data were organized and pre‐processed using Microsoft Excel 2019 (Microsoft, Redmond, WA, USA). Inter‐group differences in α‐diversity indices and continuous variables were analysed using SPSS Statistics 27.0 (IBM, Armonk, NY, USA). For data meeting parametric assumptions, one‐way analysis of variance (one‐way ANOVA) was performed to test overall inter‐group differences, followed by Tukey's honestly significant difference (HSD) post hoc test for pairwise multiple comparisons at a significance level of *α* = 0.05. Inter‐group differences in β‐diversity were evaluated via permutational multivariate analysis of variance (PERMANOVA) in R v4.3.2 (R Foundation for Statistical Computing, Vienna, Austria). All remaining figures were prepared using Origin 2024 (OriginLab Corporation, Northampton, MA, USA).

#### 2.2.7 Ethical Statement

The kelp gametophyte germplasm used in this study is a common laboratory strain and is not an endangered or protected species. All experimental procedures were conducted on plant and microbial materials under laboratory conditions and did not involve any experimentation on animals. This research was conducted in accordance with standard academic ethical guidelines.

#### 2.2.8 Definitions of Abbreviations Used in This Study

Table 1 lists the abbreviations used in this study and their corresponding descriptions. The abbreviations are divided into two categories: Antibiotic Treatment (KS1 and KS2) and Temperature Treatments (PZ1, WK1, WK2 and WK3), detailing the experimental groups for antibiotic conditions and different culture temperatures.

**TABLE 1:** Abbreviations used in this study.

Category	Abbreviation	Description
Antibiotic treatment	KS1	Group without antibiotic addition
	KS2	Group with antibiotic addition
Temperature treatments	PZ1	Epiphytic bacteria of kelp gametophytes
	WK1	Planktonic bacteria cultured at 10°C
	WK2	Planktonic bacteria cultured at 15°C
	WK3	Planktonic bacteria cultured at 20°C

## 3 Results and Analysis

### 3.1 Effects of Antibiotic on Phycosphere Bacterial Communities in Kelp Gametophytes

#### 3.1.1 Species Richness Analysis Under Antibiotic Treatment

Following sequencing on the Illumina MiSeq PE300 platform and stringent quality control filtration, high‐fidelity sequencing data were obtained for downstream analyses. Post‐normalization analysis of diversity revealed that (Figure 1) identified 27 conserved operational taxonomic units present across all experimental cohorts, delineating a core microbiome. The control group KS1 contained 104 OTUs in total, with 77 unique to this group, whereas the antibiotic‐treated group KS2 contained 81 OTUs in total, with 54 unique. Comparative analysis revealed distinct restructuring between these communities, evidenced by significant divergences in sample‐specific unique OTU composition (*p* < 0.05). To further quantify community restructuring, alpha diversity metrics were analysed.

**FIGURE 1:**
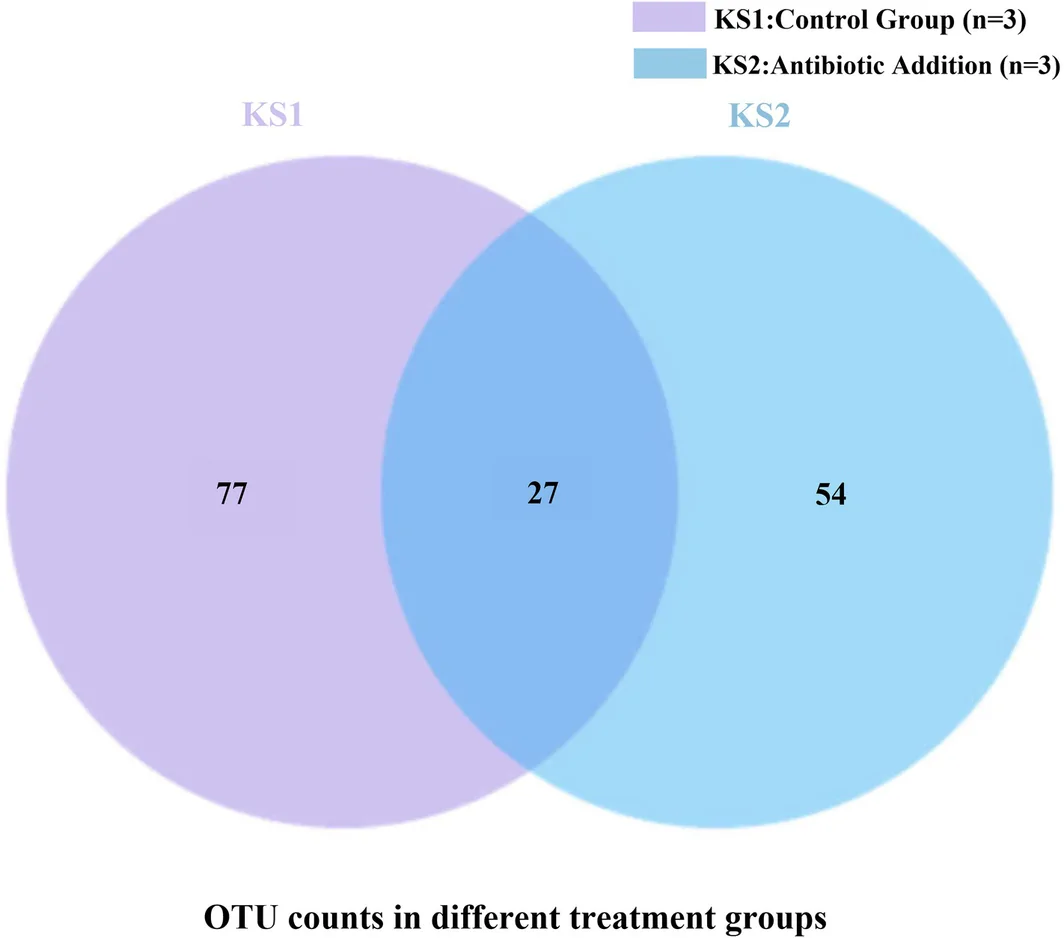
OTU distribution across treatment groups. Venn diagram showing the distribution of operational taxonomic units (OTUs) in the control group (KS1) and antibiotic addition group (KS2). The numbers represent the count of unique or shared OTUs. (*n* = 3 per group).

The Shannon index was significantly higher in KS2 than in KS1 (Figure 2a), with KS1 showing a constrained interquartile range and KS2 an expanded one, indicating greater inter‐sample heterogeneity. The Chao1 and ACE indices, which quantify species richness, were significantly greater in KS1 than in KS2 (Figure 2b,c), consistent with the reduction in total and unique OTU counts. The Simpson index was significantly higher in KS2 (Figure 2d), reflecting greater dominance by specific bacterial taxa in the antibiotic‐treated group.

**FIGURE 2:**
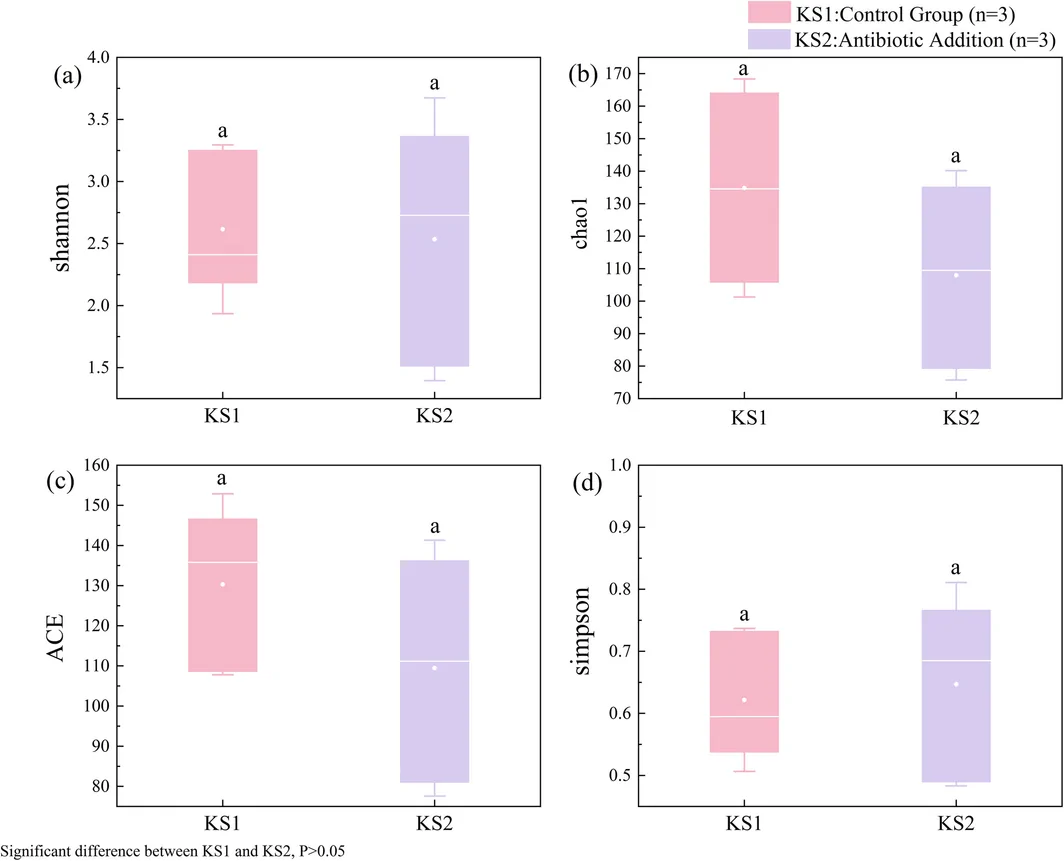
Alpha diversity across treatment groups. (a) Shannon index. (b) Chao1 index. (c) ACE index. (d) Simpson index. Box plots show the distribution of alpha diversity metrics for each group. In the box plots, the horizontal line represents the median and the dot represents the mean. Different lowercase letters indicate no significant difference between groups. KS1: Control Group; KS2: Antibiotic Addition.

These results correspond to antibiotic‐mediated community simplification: antimicrobial agents selectively eliminate susceptible taxa, reducing overall richness while allowing resistant populations to increase in relative abundance.

#### 3.1.2 PCoA Analysis

Principal Coordinates Analysis (PCoA) of antibiotic‐treated (KS2) and control (KS1) cohorts is presented in Figure 3 PCoA axes 1 and 2 accounted for 42.2% and 32.29% of the total variance, respectively, capturing the major axes of inter‐sample β‐diversity. Distinct spatial clustering patterns separated KS1 and KS2 cohorts along the PCoA1 axis. PERMANOVA indicated that this community differentiation did not reach statistical significance at *α* = 0.05 (*p* = 0.1, *R* ^2^ = 0.4), a result likely attributable to the limited sample size (*n* = 3 per group), which constrains the statistical power of the test. Nevertheless, the relatively high *R* ^2^ value and the clear visual separation along PCoA1 suggest a biologically relevant trend of community divergence attributable to antibiotic intervention, warranting further investigation with expanded replication.

**FIGURE 3:**
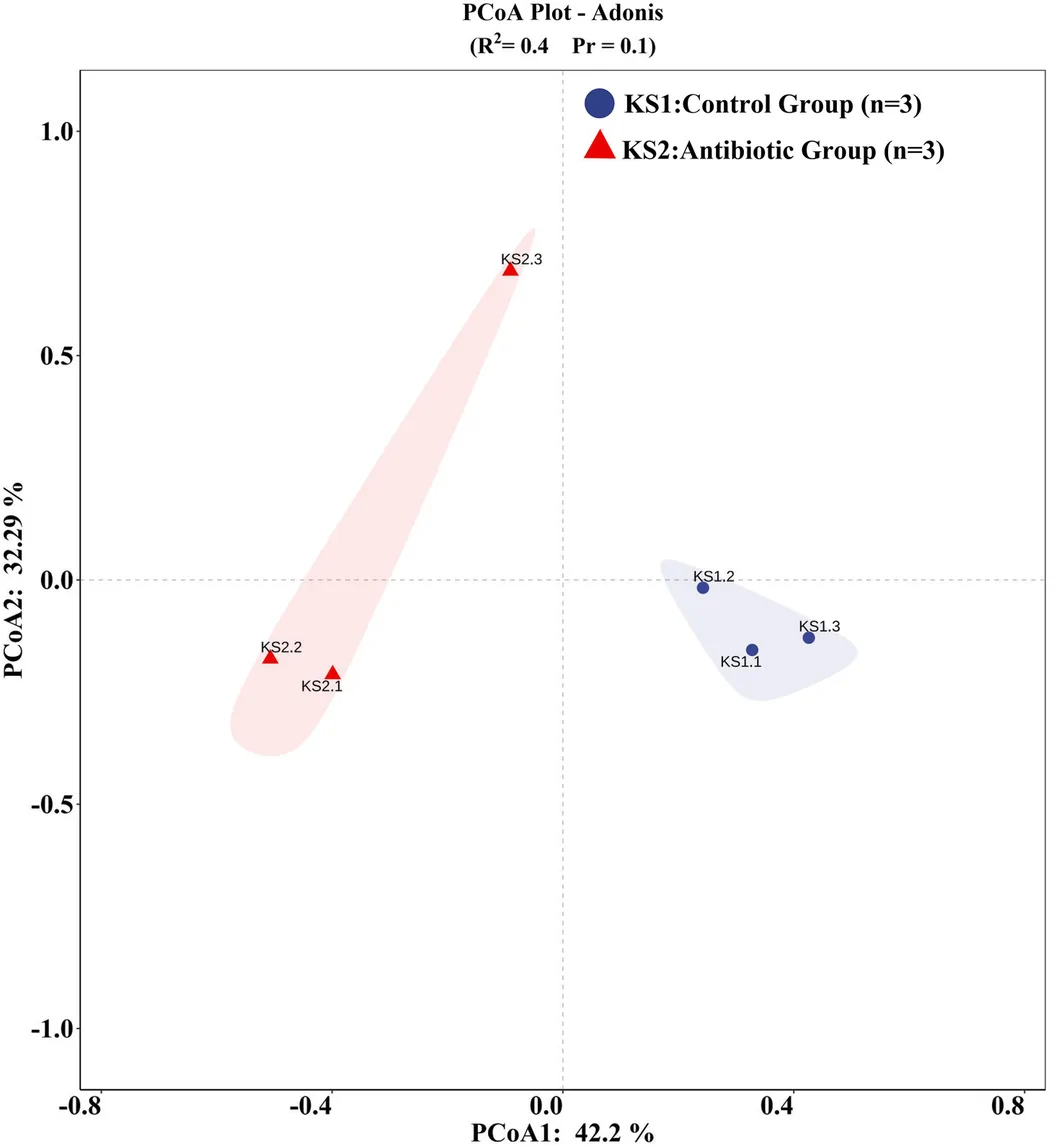
PCoA analysis across treatment groups. Blue circles represent the control group (KS1), and red triangles represent the antibiotic‐treated group (KS2). The percentages on the axes indicate the variance explained by each principal coordinate. Adonis (PERMANOVA) test was performed to evaluate the significance of group differences. *R* ^2^ indicates the proportion of variance explained by the grouping factor, and *p*_r_ represents the significance (*p*‐value).

### 3.2 Effects of Temperature on Phycosphere Communities

#### 3.2.1 Specific Growth Rate

Specific growth rate quantifies growth velocity within defined time intervals. As presented in Figure 4, kelp gametophytes exhibited maximal specific growth at 15°C (5.21% d^−1^), significantly exceeding rates observed at 10°C (3.20% d^−1^) and 20°C (3.95% d^−1^). This temperature‐dependent growth pattern identifies 15°C as the thermal optimum for gametophyte development, while the 34.7% reduction at 10°C relative to the 15°C optimum signifies pronounced cold‐temperature growth limitation.

**FIGURE 4:**
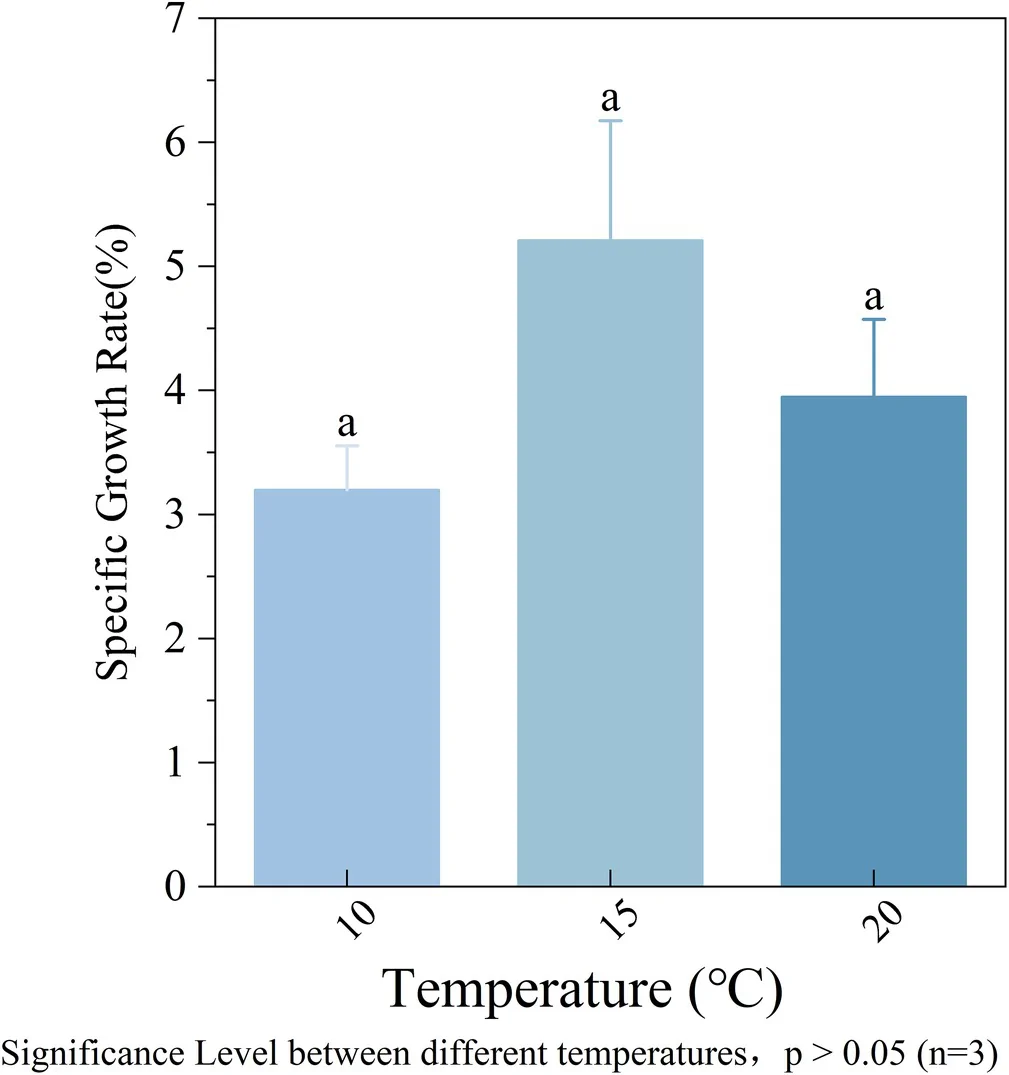
Specific growth rate. The bar chart shows the specific growth rate (%) at 10°C, 15°C, and 20°C. Data are presented as mean ± standard error/deviation (*n* = 3). Bars sharing the same lowercase letter indicate no significant difference among temperatures (*p* > 0.05).

#### 3.2.2 Species Richness Analysis Under Different Temperature Treatments

Following sequencing on the Illumina MiSeq PE300 platform and stringent quality control filtration, high‐fidelity sequencing data were obtained. Subsequent to normalization, analysis of its diversity revealed that (Figure 5) identified 8 conserved operational taxonomic units present across all experimental cohorts, delineating a distinct core microbiome. The epiphytic bacteria group PZ1 contained 74 OTUs in total, with 48 unique to this group, whereas the planktonic bacteria groups WK1, WK2, and WK3 contained 80, 70, and 51 OTUs in total, with 62, 43, and 29 unique OTUs, respectively. Comparative analysis revealed distinct restructuring between these communities, evidenced by divergences in sample‐specific unique OTU composition. To further quantify community restructuring, alpha diversity metrics were analysed.

**FIGURE 5:**
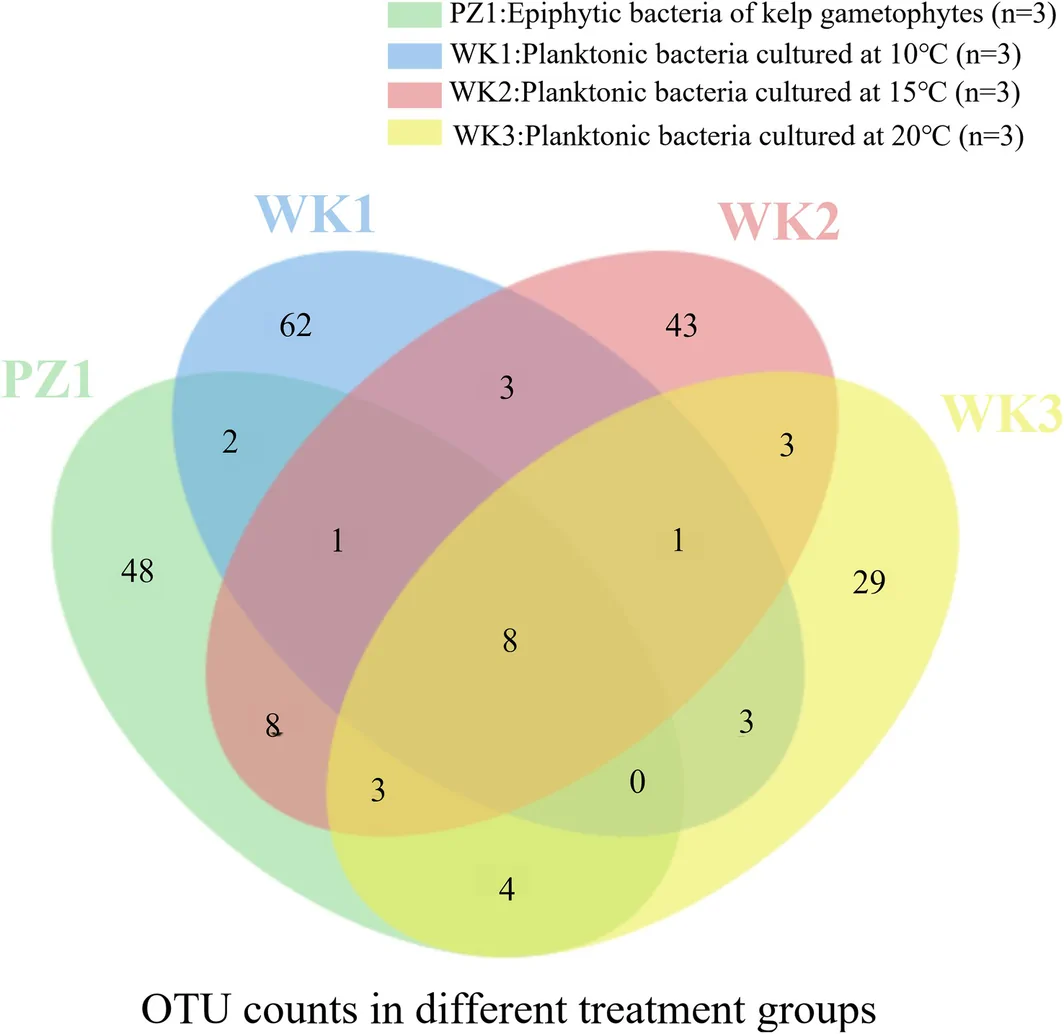
OTU distribution across temperature regimes. Venn diagram showing the distribution of operational taxonomic units (OTUs) in the epiphytic bacteria group (PZ1) and planktonic bacteria groups under different temperature regimes (WK1, WK2, WK3). The numbers represent the count of unique or shared OTUs (*n* = 3 per group).

The Shannon index showed no significant difference among PZ1, WK1, WK2, and WK3 (Figure 6a), although WK3 exhibited a diminished median index with expanded whisker ranges, suggesting reduced diversity metrics and pronounced inter‐sample heterogeneity. The Chao1 and ACE indices, which quantify species richness, showed lower values in WK3 compared to PZ1, WK1, and WK2 (Figure 6b,c), consistent with the reduction in total and unique OTU counts observed in the Venn diagram. The Simpson index (Figure 6d) was elevated in WK3, reflecting greater dominance by specific bacterial taxa under elevated thermal regimes.

**FIGURE 6:**
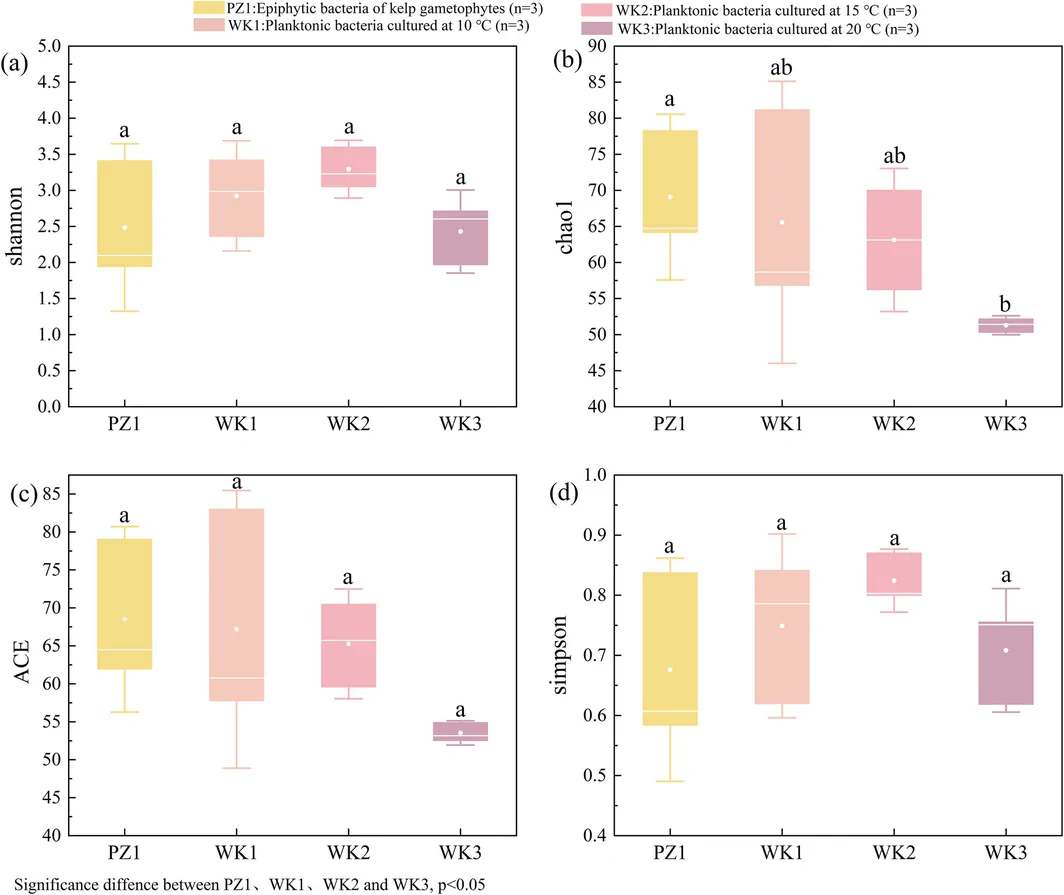
Alpha diversity across temperature regimes. (a) Shannon index. (b) Chao1 index. (c) ACE index. (d) Simpson index. Box plots show the distribution of alpha diversity metrics for each group. In the box plots, the horizontal line represents the median and the dot represents the mean. Different lowercase letters indicate differences between groups. PZ1: Epiphytic bacteria of kelp gametophytes; WK1, WK2, and WK3: Planktonic bacteria cultured at 10°C, 15°C, and 20°C, respectively (*n* = 3 per group).

These results correspond to temperature‐mediated community restructuring: cultures maintained at 20°C generated the lowest OTU richness indices, indicating that elevated thermal regimes suppress species diversity and reduce overall richness while allowing thermotolerant populations to increase in relative abundance. Conversely, suboptimal temperatures (10°C) sustained multispecies coexistence and maintained richness comparable to PZ1 controls.

#### 3.2.3 PCoA Analysis

Principal Coordinates Analysis (PCoA) of planktonic bacterial communities associated with *S. japonica* gametophytes across thermal regimes is presented in Figure 7. PCoA axes 1 (46.43%) and 2 (22.55%) collectively accounted for 69.98% of total β‐diversity variation, effectively resolving inter‐sample dissimilarities. Distinct spatial clustering patterns emerged among the four cohorts. WK1 and WK2 formed tightly aggregated clusters (PERMANOVA: *p* = 0.116, *R* ^2^ = 0.378), indicating compositional homogeneity within these cold‐adapted thermal groups and suggesting the existence of a shared thermal niche for planktonic bacteria under suboptimal temperature conditions. In contrast, WK3 and PZ1 exhibited marked spatial separation from the cooler groups, reflecting a temperature‐driven ecological niche partitioning. Specifically, the 20°C regime induced a substantial restructuring of the planktonic microbiome, while PZ1 occupied a distinct ecological niche that diverged markedly from both the cold‐adapted consortia and the general 20°C planktonic background. While the PERMANOVA result did not reach statistical significance at *α* = 0.05, the relatively high R^2^ value and the clear ordination pattern suggest a biologically meaningful thermal niche differentiation among planktonic bacterial assemblages.

**FIGURE 7:**
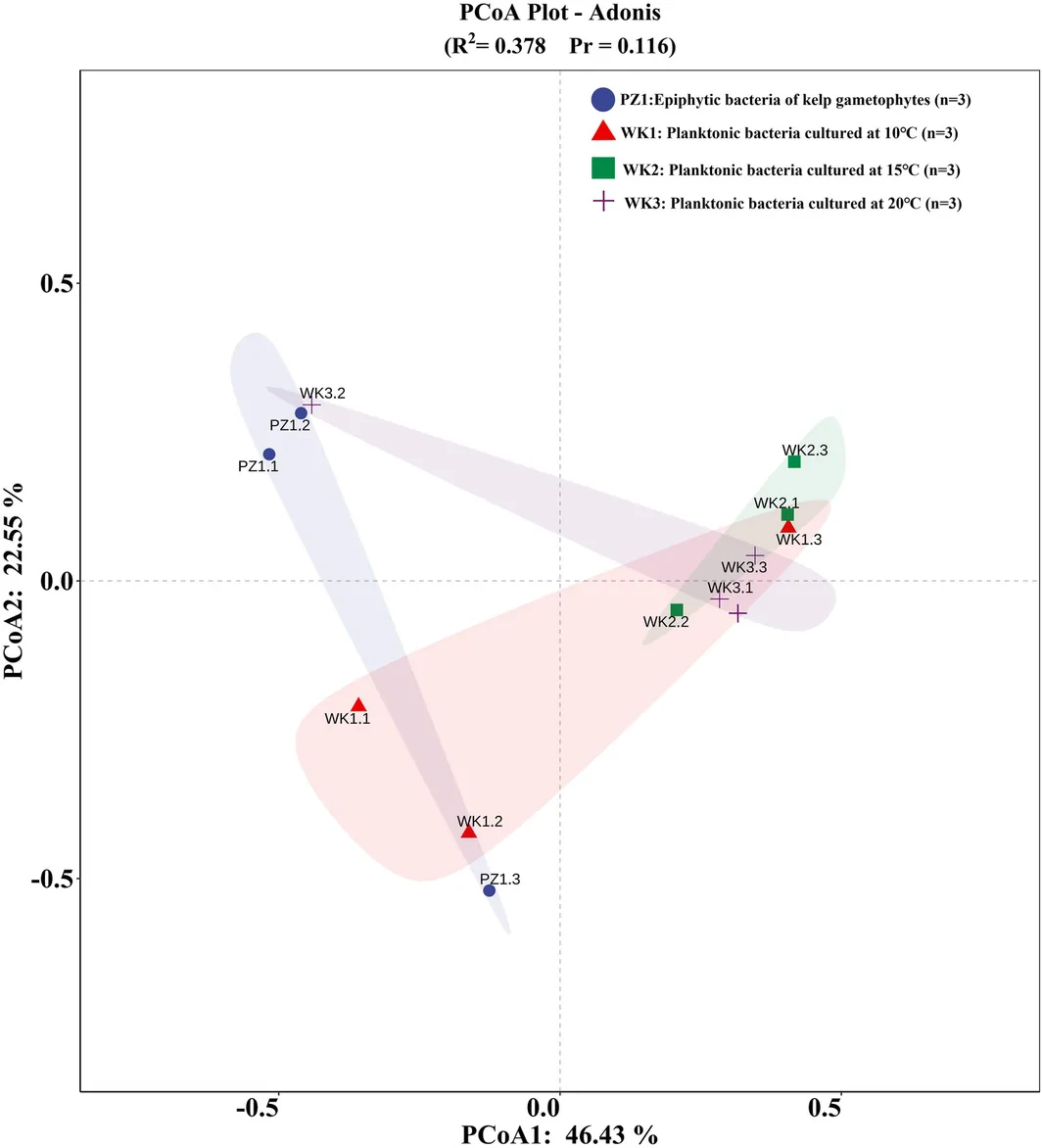
PCoA analysis across temperature regimes. Blue circles represent PZ1 (Epiphytic bacteria of kelp gametophytes); red triangles represent WK1 (Planktonic bacteria cultured at 10°C); green squares represent WK2 (Planktonic bacteria cultured at 15°C); purple crosses represent WK3 (Planktonic bacteria cultured at 20°C). The percentages on the axes indicate the variance explained by each principal coordinate. Adonis (PERMANOVA) test was performed to evaluate the significance of group differences. *R* ^2^ indicates the proportion of variance explained by the grouping factor, and *p*_r_ represents the significance (*p*‐value). (*n* = 3 per group).

#### 3.2.4 Clustering Tree Analysis

Clustering dendrograms delineate microbial sample relationships through hierarchical algorithms based on beta‐diversity metrics. The dendrogram in Figure 8 illustrates phylogenetic relationships among experimental cohorts, with closer branch proximity indicating greater compositional congruence in bacterial community structure.

**FIGURE 8:**
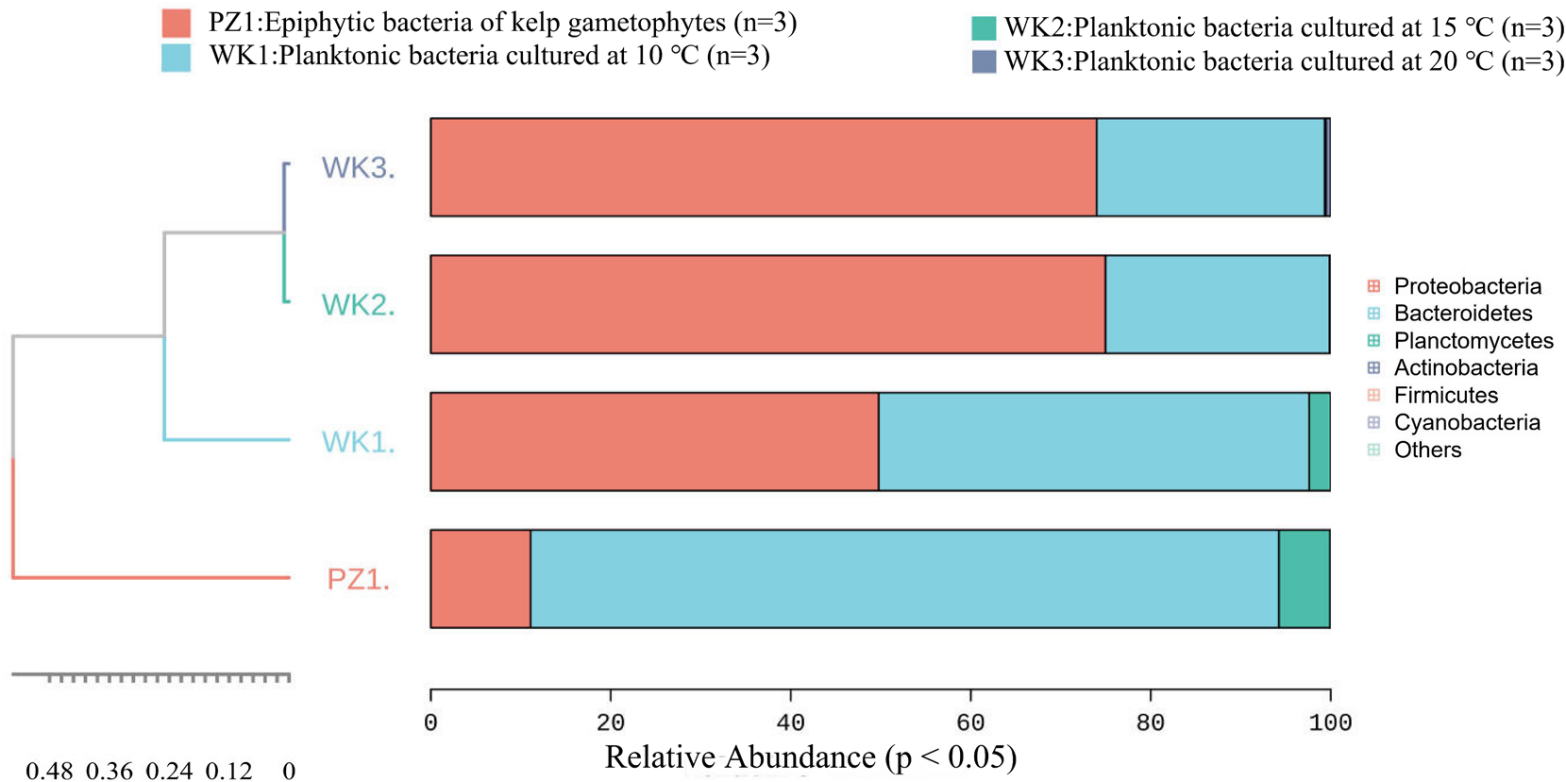
UPGMA clustering tree analysis. The hierarchical clustering tree on the left shows the similarity of bacterial community composition among groups. Only phyla with relative abundance > 0.05 are shown. Red represents PZ1 (Epiphytic bacteria of kelp gametophytes); light blue represents WK1 (Planktonic bacteria cultured at 10°C); green represents WK2 (Planktonic bacteria cultured at 15°C); dark blue represents WK3 (Planktonic bacteria cultured at 20°C). (*n* = 3 per group).

Hierarchical clustering demonstrated distinct phylogenetic groupings, with WK1, WK2, and WK3 forming a cohesive cluster indicative of shared community composition. PZ1 constituted a phylogenetically distant clade exhibiting fundamentally divergent community architecture. At the phylum level, Bacteroidetes accounted for over 85% of epiphytic PZ1 sequences. Proteobacteria dominated WK1, with abundance significantly higher than Bacteroidetes (*p* < 0.05) and low‐abundance Actinobacteria detected. WK2 assemblages showed co‐dominance of Proteobacteria and Bacteroidetes with residual Actinobacteria. WK3 was dominated by Proteobacteria, whose abundance exceeded that of Bacteroidetes (*p* < 0.01), with Firmicutes as a minor phylum.

While WK cohorts exhibited conserved clustering topology, significant differential abundance profiles emerged at the phylum level. PZ1 demonstrated exclusive Bacteroidetes predominance (*p* < 0.01), contrasting fundamentally with the conserved Proteobacterial predominance observed across all temperature‐treated WK groups.

### 3.3 Effects of Different Stress Conditions on the Phycosphere Microbiota of Kelp Gametophytes

#### 3.3.1 Phylum‐Level Analysis

Figure 9a, by comparing the control group (KS1) with the antibiotic‐treated group (KS2), clearly demonstrates the strong selective pressure exerted by antibiotics on the bacterial community. In the control group KS1, Proteobacteria was the absolutely dominant phylum, exhibiting a very high relative abundance. In contrast, in the antibiotic‐treated group KS2, the abundance of this phylum decreased significantly, indicating a substantial inhibitory effect of the antibiotics on its growth. As the abundance of Proteobacteria declined, opportunities for other bacterial phyla increased. The most pronounced change was observed in Bacteroidetes, whose relative abundance increased sharply in KS2 compared to KS1, becoming a major phylum with abundance comparable to that of Proteobacteria.

**FIGURE 9:**
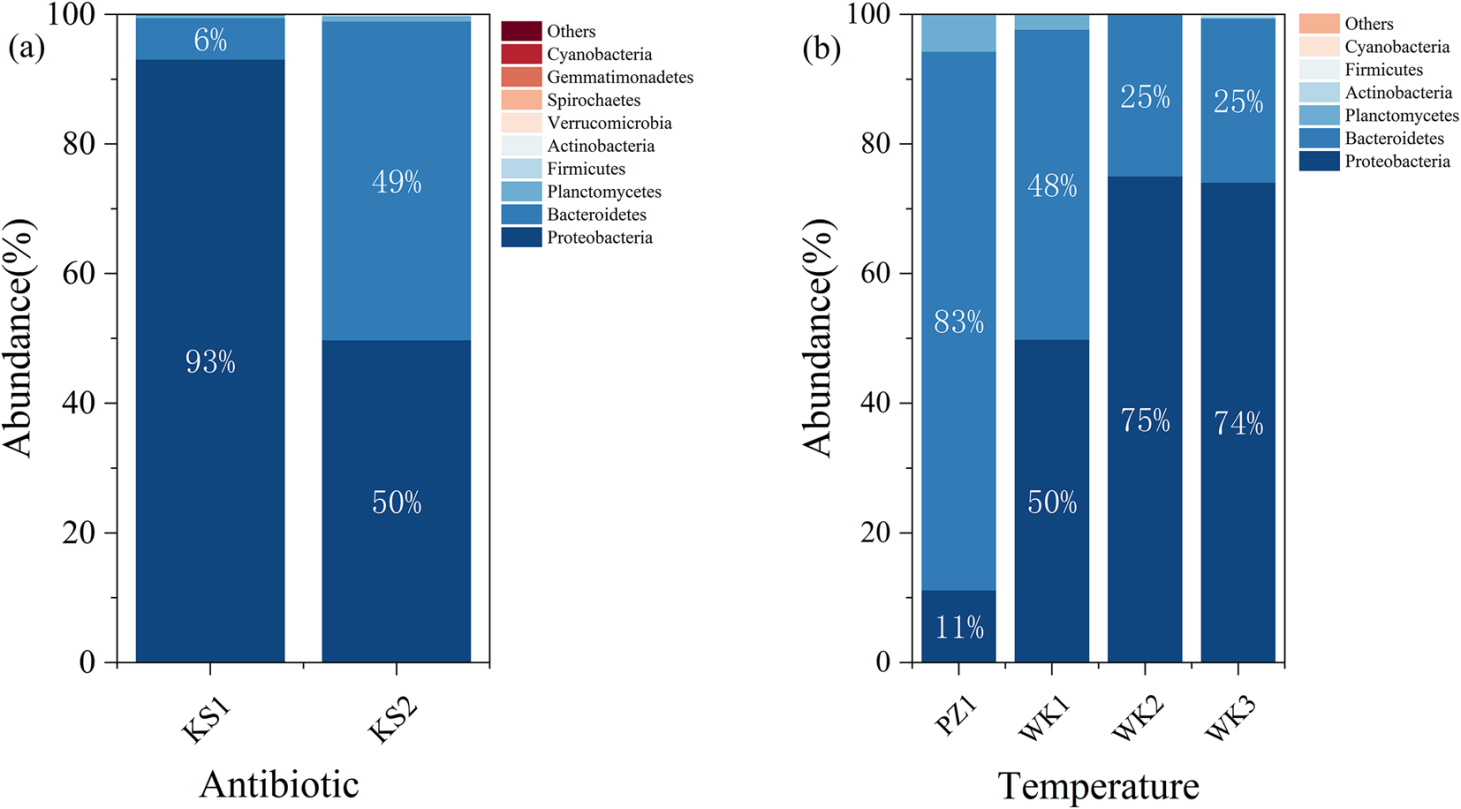
Major bacterial phyla under different conditions. (a) Relative abundance of major bacterial phyla in the control group (KS1) and antibiotic‐treated group (KS2). (b) Relative abundance of major bacterial phyla in epiphytic bacteria of kelp gametophytes (PZ1) and planktonic bacteria cultured at 10°C (WK1), 15°C (WK2), and 20°C (WK3). Only phyla with relative abundance > 0.05 are shown. (*n* = 3 per group).

As shown in Figure 9b, in contrast to the drastic shifts induced by antibiotic treatment, the composition of dominant phyla remained relatively stable across samples subjected to different temperature conditions. Proteobacteria maintained a high relative abundance in all temperature samples, indicating its broad temperature adaptability and satisfactory acclimation to the experimental temperature gradient. Although the dominant phylum remained unchanged, the relative proportions of the various phyla exhibited regular variations in response to temperature. Bacteroidetes dominated the epiphytic PZ1 assemblage but declined progressively within the planktonic fraction as temperature increased, co‐dominating with Proteobacteria at 15°C and being supplanted by Proteobacteria at 20°C.

#### 3.3.2 Genus‐Level Analysis

Figure 10a, by comparing the control group (KS1) and the antibiotic‐treated group (KS2), reveals significant shifts in the bacterial community at the genus level. In the control group KS1, genera such as *Colwellia* and *Sulfitobacter* were the most dominant taxa. However, in the antibiotic‐treated group KS2, the relative abundances of these genera decreased sharply, indicating their high sensitivity to the antibiotics. Meanwhile, certain genera that were present in very low abundances or undetectable in KS1 became the new dominant taxa in KS2. For instance, the relative abundances of genera such as *Kordia* and Klebsiella increased significantly in KS2, demonstrating their strong tolerance or resistance to the applied antibiotics.

**FIGURE 10:**
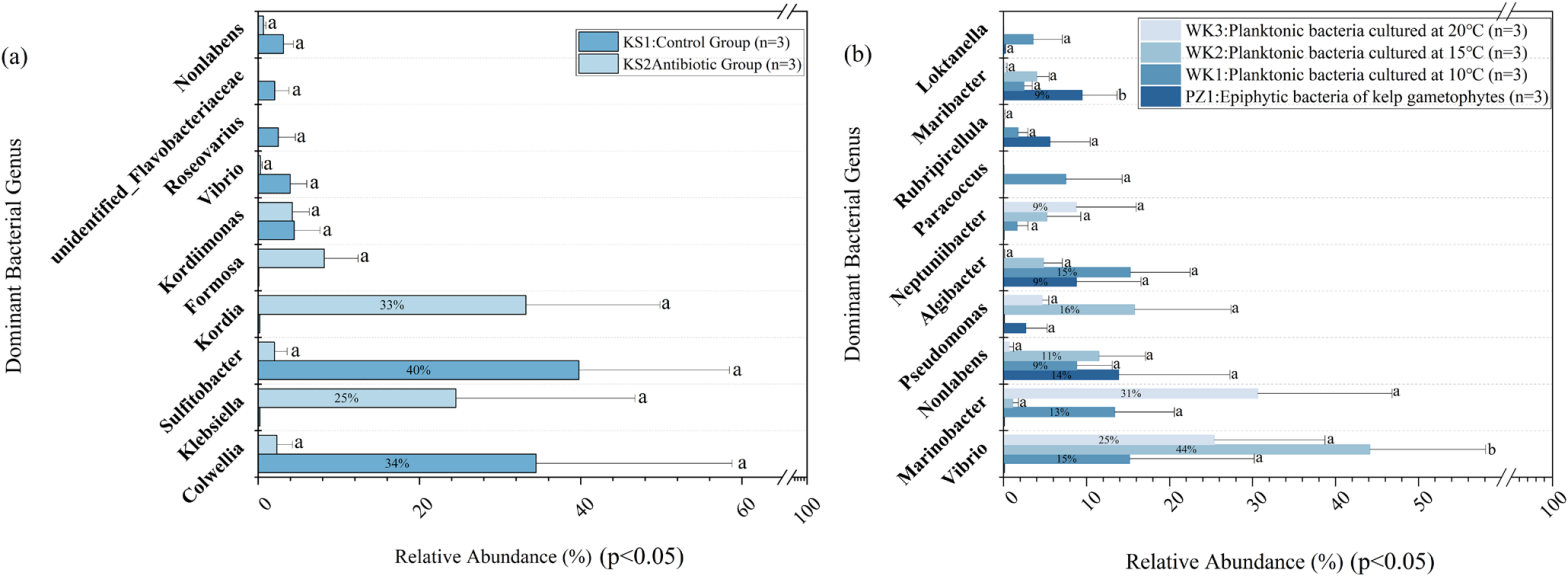
Major bacterial genera under different conditions. (a) Relative abundance of dominant bacterial genera in the control group (KS1) and antibiotic‐treated group (KS2). (b) Relative abundance of dominant bacterial genera in epiphytic bacteria of kelp gametophytes (PZ1) and planktonic bacteria cultured at 10°C (WK1), 15°C (WK2), and 20°C (WK3). Only genera with relative abundance > 0.05 are shown. Different lowercase letters indicate differences between groups. (*n* = 3 per group).

Figure 10b illustrates community composition across the temperature treatments., reflecting the continuous regulatory effect of temperature on community composition. In contrast to the drastic changes induced by antibiotic treatment, Temperature induced quantitative fluctuations in taxon abundances without driving qualitative turnover of dominant community members. Genus‐level analysis revealed temperature‐dependent distribution patterns. At 10°C, no single genus exhibited absolute dominance, though *Algibacter* (15.26%) and *Vibrio* (15.16%) demonstrated comparatively higher prevalence. Cultures maintained at 15°C displayed pronounced *Vibrio* predominance (44.11%). Under 20°C conditions, *Marinobacter* (30.61%) and *Vibrio* (25.35%) emerged as co‐dominant taxa. *Vibrio* manifested significant differential abundance at 20°C compared to other cohorts (*p* < 0.05), while *Maribacter* at 10°C exhibited statistically distinct abundance profiles (*p* < 0.05).

## 4 Conclusions and Discussion

### 4.1 Impact of Antibiotic Treatments on the Bacterial Community Structure in the Phycosphere of Kelp Gametophytes

Penicillin–streptomycin treatment reduced Proteobacteria from 93.02% to 49.70% and enriched Bacteroidetes from 6.98% to 49.20%, with a genus‐level shift from *Sulfitobacter* and *Colwellia* to *Kordia* and *Klebsiella*.

At the phylum level, the reduction of Proteobacteria and the concomitant enrichment of Bacteroidetes are consistent with the known targets of penicillin and streptomycin and with differences in growth strategy and envelope permeability. Penicillin inhibits peptidoglycan cross‐linking and streptomycin impairs translation, which together preferentially suppress fast‐growing surface‐associated Proteobacteria that rely on sustained biosynthesis to maintain epiphytic populations (Moazed and Noller 1987). In contrast, Bacteroidetes exhibit relative tolerance linked to outer‐membrane‐mediated permeability control and slower growth, and they can increase in relative abundance when competitors are suppressed (Hedberg and Nord 1996). Ecologically, Proteobacterial clades create vacant niches and reduce competitive exclusion for nutrient resources. Concurrently, Bacteroidetes can exploit carbon and nitrogen substrates released from lysed sensitive cells (Chabanol et al. 2020), providing an immediate post‐disturbance growth advantage.

At the genus level, the loss of *Sulfitobacter* and *Colwellia* and the enrichment of *Kordia* and *Klebsiella* indicate a functional turnover in the epiphytic consortium. *Sulfitobacter* and *Colwellia* have been reported to engage in mutualistic interactions with marine algae and can contribute to suppression of opportunistic colonizers through competitive exclusion and production of antimicrobial metabolites; this refers to protection of the host rather than degradation of applied antibiotics (Amin et al. 2015). Their reduction under antibiotic treatment suggests a weakening of the native microbial barrier on kelp gametophyte surfaces. *Kordia* includes Bacteroidetes with polysaccharide‐degrading capacities that enable growth on algal‐derived substrates, which may confer a post‐disturbance advantage (Lim et al. 2020). In the context of long‐term germplasm preservation, the proliferation of polysaccharide‐degrading *Kordia* may alter the physicochemical properties of the phycosphere mucus layer, potentially affecting gametophyte surface integrity during extended storage. *Klebsiella* is an opportunistic taxon with documented resistance traits in other systems. Its increase here should be interpreted cautiously as an indicator of community dysbiosis rather than evidence of pathogenicity. The enrichment of *Klebsiella* in antibiotic‐treated samples raises concerns regarding the proliferation of antimicrobial resistance genes within the preservation system, particularly if antibiotic residues persist.

In relation to kelp preservation, the observed phylum‐ and genus‐level shifts are consistent with a transition from a community that supports colonization resistance to one with reduced barrier function. Because prophylactic antibiotic use did not lower disease risk in our system and coincided with enrichment of opportunistic or disturbance‐favoured taxa, careful evaluation of antimicrobial interventions in germplasm culture is warranted.

### 4.2 Temperature Effects on Phycosphere Communities of Kelp Gametophytes

Temperature variation across the 10°C–20°C range induced quantitative restructuring of the gametophyte phycosphere without triggering wholesale taxonomic turnover. Within this tolerated thermal envelope, temperature acts as a metabolic modulator that adjusts the growth rates and carrying capacities of major bacterial phylogenetic groups, producing abundance shifts without displacing core taxa (Arandia‐Gorostidi et al. 2017). Consistent with observations from other kelp systems where gradual shifts occur within thermal tolerance limits (Molnar et al. 2025). the present gametophyte community remained stable across 10°C–20°C, a range far below the approximately 26°C threshold associated with wholesale collapse (Veenhof et al. 2025). Thus, temperature modulated competitive hierarchies while the ecological filters governing phycosphere colonization stayed intact.

At 15°C, the gametophyte specific growth rate peaked at 5.21% d^−1^, while pathogenic *Vibrio* and *Pseudomonas* concurrently reached their maximum relative abundances. Below 15°C, such as at 10°C, growth rate decreases but the risk of pathogenic invasion is reduced, resulting in a more stable epiphytic community. Above 15°C, such as at 20°C, gametophyte growth rate declines and thermal stress may compromise host health, even though the relative abundance of certain pathogens might decrease. For kelp preservation, this creates a trade‐off: 15°C maximizes biomass accumulation but coincides with the highest contamination risk, whereas lower temperatures like 10°C minimize microbial disturbance at the cost of slower growth. Therefore, the choice of temperature should align with the preservation objective.

These findings highlight a fundamental dichotomy in how antibiotics and temperature regulate epiphytic communities, with direct implications for the design of germplasm preservation protocols.

### 4.3 Implications for Germplasm Preservation

Based on the relationships between treatment, microbial community shifts, and host health, the following strategies are proposed to improve the quality of kelp germplasm preservation. For antibiotic use, the observed qualitative community turnover, characterized by the loss of beneficial mutualists (*Sulfitobacter*, *Colwellia*) and the enrichment of opportunistic taxa (*Kordia*, *Klebsiella*), indicates that broad‐spectrum antibiotics disrupt the native microbial barrier and create unoccupied niches that facilitate dysbiosis. Chronic application further risks selecting for antimicrobial‐resistant strains. Therefore, prophylactic antibiotic use should be avoided in germplasm preservation protocols. If contamination control is necessary, short‐term, low‐dose, or taxon‐selective interventions are recommended over broad‐spectrum suppression, and only during the initial culture phase rather than long‐term storage. For temperature management, the quantitative restructuring driven by temperature directly modulates pathogenic loads without displacing core taxa. At 15°C, the thermal optimum for gametophyte growth coincides with peak relative abundances of *Vibrio* (44.11%) and *Pseudomonas* (15.77%), meaning that conditions favouring biomass accumulation simultaneously elevate disease risk. For short‐term seedling production where rapid growth is prioritized, cultures can be maintained at 15°C, but this requires concurrent microbial monitoring or aseptic handling to suppress pathogen proliferation. Conversely, at 10°C, pathogen abundances are significantly lower and community structure and alpha diversity remain stable. For long‐term germplasm conservation, constant storage at 10°C without antibiotic supplementation is advised. This approach preserves the integrity of the native epiphytic microbiome, maintains colonization resistance against opportunistic invaders, and avoids antibiotic‐induced dysbiosis, thereby improving the viability and quality of preserved gametophytes.

## Author Contributions

**Yuewen Zhang:** conceptualization, software, writing – original draft, methodology. **Shanshan Liang:** conceptualization, writing – original draft. **Huiling Xia:** validation, supervision. **Xiaoyu Zhong:** investigation. **Yi Zheng:** conceptualization, writing – review and editing, supervision, data curation.

## Funding

This work was supported by the Regional Development Project of Fujian Province  (2017N3015) and Demonstration Project on Innovative Development of Marine Economy in the 13th Five‐Year Plan of the State Oceanic Administration (FZHJ03).

## Conflicts of Interest

The authors declare no conflicts of interest.

## Data Availability

The data presented in the study are deposited in the NCBI Short Read Archive (SRA), accession numbers PRJNA1506064.

## References

[emi470425-bib-0001] Amin, S. A. , L. R. Hmelo , H. M. Van Tol , et al. 2015. “Interaction and Signalling Between a Cosmopolitan Phytoplankton and Associated Bacteria.” Nature 522, no. 7554: 98–101. 10.1038/nature14488. 26017307

[emi470425-bib-0002] Arandia‐Gorostidi, N. , T. M. Huete‐Stauffer , L. Alonso‐Sáez , and X. A. G. Morán . 2017. “Testing the Metabolic Theory of Ecology With Marine Bacteria: Different Temperature Sensitivity of Major Phylogenetic Groups During the Spring Phytoplankton Bloom.” Environmental Microbiology 19, no. 11: 4493–4505. 10.1111/1462-2920.13898. 28836731

[emi470425-bib-0003] Bell, W. , and R. Mitchell . 1972. “Chemotactic and Growth Responses of Marine Bacteria to Algal Extracellular Products.” Biological Bulletin 143, no. 2: 265–277. 10.2307/1540052.

[emi470425-bib-0005] Castro, L. C. , A. Vergés , S. C. Straub , et al. 2024. “Effect of Marine Heatwaves and Warming on Kelp Microbiota Influence Trophic Interactions.” Molecular Ecology 33, no. 5: e17267. 10.1111/mec.17267. 38230446

[emi470425-bib-0006] Chabanol, E. , V. Behrends , G. Prévot , G. K. Christophides , and M. Gendrin . 2020. “Antibiotic Treatment in Anopheles Coluzzii Affects Carbon and Nitrogen Metabolism.” Pathogens 9, no. 9: 679. 10.3390/pathogens9090679. PMC7558193 32825534

[emi470425-bib-0007] Citti, C. , and A. Blanchard . 2013. “Mycoplasmas and Their Host: Emerging and Re‐Emerging Minimal Pathogens.” Trends in Microbiology 21, no. 4: 196–203. 10.1016/j.tim.2013.01.003. 23419218

[emi470425-bib-0010] Hedberg, M. , and C. E. Nord . 1996. “Beta‐Lactam Resistance in Anaerobic Bacteria: A Review.” Journal of Chemotherapy 8, no. 1: 3–16. 10.1179/joc.1996.8.1.3. 8835102

[emi470425-bib-0012] Lee, J.‐H. , D.‐H. Kim , S.‐P. Jung , S.‐J. Choi , I.‐K. Chung , and J.‐A. Shin . 2005. “Cultivation of Laminaria Japonica (Laminariales, Phaeophyta) in Udo Coast, Jeju, Korea.” Algae 20, no. 2: 167–176. 10.4490/ALGAE.2005.20.2.167.

[emi470425-bib-0013] Lian, J. , R. H. Wijffels , H. Smidt , and D. Sipkema . 2018. “The Effect of the Algal Microbiome on Industrial Production of Microalgae.” Microbial Biotechnology 11, no. 5: 806–818. 10.1111/1751-7915.13296. PMC6116740 29978601

[emi470425-bib-0014] Lim, Y. , I. Kang , and J.‐C. Cho . 2020. “Genome Characteristics of Kordia Antarctica IMCC3317T and Comparative Genome Analysis of the Genus Kordia.” Scientific Reports 10, no. 1: 14715. 10.1038/s41598-020-71328-9. PMC7477175 32895436

[emi470425-bib-0015] Mars Brisbin, M. , A. Schofield , M. R. McIlvin , A. I. Krinos , H. Alexander , and M. A. Saito . 2023. “Vitamin B12 Conveys a Protective Advantage to Phycosphere‐Associated Bacteria at High Temperatures.” ISME Communications 3, no. 1: 88. 10.1038/s43705-023-00298-6. PMC10457287 37626172

[emi470425-bib-0017] Moazed, D. , and H. F. Noller . 1987. “Interaction of Antibiotics With Functional Sites in 16S Ribosomal RNA.” Nature 327, no. 6121: 389–394. 10.1038/327389a0. 2953976

[emi470425-bib-0018] Molnar, N. B. , B. L. Weigel , R. J. Fales , and C. A. Pfister . 2025. “Warming Seawater Temperature and Nutrient Depletion Alters Microbial Community Composition on a Foundational Canopy Kelp Species.” Environmental Microbiology 27, no. 3: e70077. 10.1111/1462-2920.70077. PMC11903912 40075558

[emi470425-bib-0019] Monteiro, C. , S. Heinrich , I. Bartsch , et al. 2019. “Temperature Modulates Sex‐Biased Gene Expression in the Gametophytes of the Kelp Saccharina Latissima.” Frontiers in Marine Science 6: 769. 10.3389/fmars.2019.00769.

[emi470425-bib-0021] Parlapani, F. F. , D. A. Anagnostopoulos , E. Karamani , A. Mallouchos , S. A. Haroutounian , and I. S. Boziaris . 2023. “Growth and Volatile Organic Compound Production of Pseudomonas Fish Spoiler Strains on Fish Juice Agar Model Substrate at Different Temperatures.” Microorganisms 11, no. 1: 189. 10.3390/microorganisms11010189. PMC9861217 36677482

[emi470425-bib-0022] Sun, F. , C. Wang , Y. Wang , K. Tu , Z. Zheng , and X. Lin . 2020. “Diatom Red Tide Significantly Drive the Changes of Microbiome in Mariculture Ecosystem.” Aquaculture 520: 734742. 10.1016/j.aquaculture.2019.734742.

[emi470425-bib-0023] Vadillo Gonzalez, S. , C. L. Hurd , D. Britton , E. Bennett , P. D. Steinberg , and E. M. Marzinelli . 2024. “Effects of Temperature and Microbial Disruption on Juvenile Kelp Ecklonia Radiata and Its Associated Bacterial Community.” Frontiers in Marine Science 10: 1332501. 10.3389/fmars.2023.1332501.

[emi470425-bib-0024] Veenhof, R. J. , M. A. Coleman , C. Champion , and S. A. Dworjanyn . 2023. “Urchin Grazing of Kelp Gametophytes in Warming Oceans.” Journal of Phycology 59, no. 5: 838–855. 10.1111/jpy.13364. 37432133

[emi470425-bib-0025] Veenhof, R. J. , A. H. McGrath , C. Champion , S. A. Dworjanyn , E. M. Marzinelli , and M. A. Coleman . 2025. “The Role of Microbiota in Kelp Gametophyte Development and Resilience to Thermal Stress.” Journal of Phycology 61, no. 3: 633–649. 10.1111/jpy.70018. PMC12168109 40299544

[emi470425-bib-0026] Wu, X. , X. Liu , S. Zhang , and Y. Zhou . 2023. “Cell Division and Meristem Dynamics in Fern Gametophytes.” Plants 12, no. 1: 209. 10.3390/plants12010209. PMC9823664 36616337

[emi470425-bib-0028] Yu, S.‐X. , L.‐Q. Hu , L.‐H. Yang , et al. 2023. “RLI2 Regulates Arabidopsis Female Gametophyte and Embryo Development by Facilitating the Assembly of the Translational Machinery.” Cell Reports 42, no. 7: 112741. 10.1016/j.celrep.2023.112741. 37421624

[emi470425-bib-0029] Zhou, Y. , W. Fang , Z. Pang , et al. 2022. “AP1G2 Affects Mitotic Cycles of Female and Male Gametophytes in Arabidopsis.” Frontiers in Plant Science 13: 924417. 10.3389/fpls.2022.924417. PMC9301471 35873977

